# CXCL13 may improve diagnosis in early neuroborreliosis with atypical laboratory findings

**DOI:** 10.1186/1471-2334-12-344

**Published:** 2012-12-10

**Authors:** Johannes P Borde, Simone Meier, Volker Fingerle, Christiane Klier, Johannes Hübner, Winfried V Kern

**Affiliations:** 1 Universitätsklinik Freiburg i.Br., Medizinische Klinik II / Sektion Klinische Infektiologie, Hugstetter Str. 55, 79106, Freiburg i.Br, Germany; 2 Ortenauklinikum Offenburg-Gengenbach, Klinik für Gastroenterologie, Hepatologie und Infektiologie, Ebertplatz 12, 77654, Offenburg, Germany; 3 Universitätsklinik Freiburg i.Br., Neurologische Klinik, Hugstetter Str. 55, 79106, Freiburg i.Br, Germany; 4 Nationales Referenzzentrum für Borrelien, Veterinärstraße 2, 85764, Oberschleißheim, Germany

**Keywords:** CXCL13, Neuroborreliosis, *Borrelia burgdorferi*, *B. garinii*, OspA-type

## Abstract

**Background:**

Current guidelines regarding Lyme neuroborreliosis [LNB] require the presence of intrathecal *Borrelia burgdorferi*-specific antibody production for the definite diagnosis of LNB. However, about 20% of early stage infections present without an elevated antibody index. Moreover, intrathecal *B. burgdorferi* specific antibody synthesis may persist long after successful therapy of LNB. Recently published data indicate that CXCL13 seems to be a promising diagnostic tool for early stage LNB. In addition, CXCL13 might be suitable for treatment monitoring.

**Case presentation:**

We report on a 39-year-old male patient from southern Germany, who has been suffering from subfebrile body temperatures and meningeal headache for six weeks. On the second day after hospital admission he developed peripheral palsy of the VII. cranial nerve. Cerebrospinal fluid (CSF) analysis showed granulocytic pleocytosis, elevated total protein and blood-CSF barrier dysfunction. Differential diagnostics for granulocytic pleocytosis were unremarkable. Only a second lumbar puncture, on day 6 after admission, revealed a lymphocytic pleocytosis. Serologic testing pointed to clear intrathecal Borrelia specific IgG antibody production. Interestingly, no anti-OspC antibodies were detectable. DNA of the rare *Borrelia garinii* OspA-type 7 could be amplified from the first CSF sample. The monitoring of CXCL13 in all CSF samples documented a fast decrease from 5000 pg/ml to 450 pg/ml after appropriate antibiotic treatment.

**Conclusion:**

CXCL13 is a novel biomarker with high sensitivity and specificity for acute LNB. Our data show, that CXCL13 might be helpful in unclear cases and support the presumption that it might be a valuable tool for treatment monitoring. Anti-OspC antibody negativity is a rare observation, given the need of OspC for infection of the human hosts. Most likely this is due to a lack of sensitivity of OspC immunoblots that are unable to detect rare OspC variants.

## Background

The multisystem disease Lyme borreliosis (LB), mainly affecting skin, nervous system and joints, is the most frequently reported vector-borne disease in the temperate zones of the Northern Hemisphere. In Europe at least five different genospecies – namely *B. burgdorferi* sensu stricto, *B. afzelii*, *B. garinii*, *B. spielmanii*, and *B. bavariensis* – may cause human diseases. The most frequent disseminated form of the disease is early Lyme-neuroborreliosis [LNB]. Current international guidelines [1] require the presence of intrathecal *B. burgdorferi*-specific antibody production for the definite diagnosis of LNB. However, about 20% of early stage infections initially present without a positive antibody index. Moreover, *B. burgdorferi* specific intrathecal antibody synthesis may persist a long time after successful therapy of LNB in terms of an “anamnestic titer” that will not allow differentiation of active from past disease. The chemokine CXCL13 (Chemokine C-X-C motif ligand 13) is known to influence homing and motility of B cells in lymphoid tissue and is involved in the formation of ectopic lymphoid tissue in chronic inflammation [2]. Recently published data [3] indicate that B cell-attracting CXCL13 seems to be a promising diagnostic tool for diagnosis of early stage LNB. In addition, CXCL13 might be suitable for the monitoring of therapy.

## Case presentation

A 39-year-old male and otherwise healthy patient presented in the emergency department with a history of 6 weeks of headache radiating to the neck and spine. The patient felt repeatedly hot flashes without reaching febrile temperature levels. On admission he also reported nausea without vomiting. The patient had no known allergies, no history of insect or animal bites, he had travelled to northern Italy 2 weeks before onset of the symptoms. One week before onset he had recieved surgical treatment of an axillary abscess under local anaesthesia. In the first two weeks of subfebrile temperatures and headache, he consulted his family doctor. Laboratory testing at his visit showed normal results for white blood-cell count and differential cell count, C-reactive protein, and serological tests for HIV, Borrelia, Yersinia, Chlamydia and Campylobacter. Results of testing for antinuclear antibodies [ANA], antineutrophil cytoplasmic antibodies [ANCA] and urinanalysis were normal. Magnetic resonance imaging [MRI] of the brain with administration of contrast material revealed no signs of inflammation, malignancies or vascular complications. The patient received an eight day therapy of steroids - 70 mg prednisolone per day – resulting in a transient improvement of his condition. However, 10 days before admission to this hospital his headache worsened again.

On admission to our hospital the temperature was 38.5°C, stable vital signs, blood pressure 140/75 mmHg, heart rate 70 beats per minute, oxygen saturation 97% while breathing ambient air. The weight was 79.5 kg. The skin was warm and dry. The abdomen was soft, normal bowel sounds, without distention, tenderness or masses. Neurological examination revealed positive meningeal signs, the remainder of the examination was normal. Echocardiography points to a mild pericardial effusion, while the electrocardiogram was normal - apart from nonspecific ST-segment changes. Standard laboratory tests were repeated, without significant findings [negative procalcitonin, negative C-reactive protein, normal white cell count]. On the second hospital day, the patient developed a right sided peripheral palsy of the VII. cranial nerve. An emergency MRI of the brain was performed, but did not show any significant pathological intracranial findings. Cerebrospinal fluid [CSF] analysis showed 280 cells, predominantly granulocytes [70%] and lymphocytes [20%], lactate and CSF total-protein levels were elevated, microscopic examination and gram staining revealed no bacteria in CSF [Table 1.]. Treatment with acyclovir [5 × 800 mg /d], ceftriaxon [2 × 2 g /d] and ampicillin [3 × 5 g /d] was promptly initiated.

**Table 1:** Cerebrospinal fluid analysis

	**1.CSF sample [day2]**	**2.CSF sample [day6]**	**3.CSF sample [day12]**	**4.CSF sample [day55]**
CSF cells [/μl]	282	364	197	18
CSF Protein [mg/l]	2470	1510	1110	673
CXCL 13 [pg/ml]	5000	2000	450	52.8
CSF lactate [mmol/l]	3.38	2.72	2.24	-
CSF lymphocytes [%]	20	80	90	-
CSF monocytes [%]	10	10	10	-
CSF granulocytes [%]	70	10	0	-

The nuclear acid testing [PCR] for HSV1, HSV2 and VZV in CSF was negative, there was no detection of enterovirus nuclear acid in the patient plasma, no clinical and laboratory signs of CNS HIV or Treponema pallidum infection were observed. CMV and EBV serology showed positive IgG titers, whereas IgM titers were negative. Additional screening for Coxiella burnetii, Brucellosis and Tuberculosis [IGRA, CSF microscopy and CSF PCR] remained negative. Borrelia serology was not done regarding predominant granulocytes in the CSF. To rule out neurosarcoidosis or systemic sarcoidosis a total body Computed Tomography [CT] was performed, but without any significant findings, in particular neopterin, soluble IL-2 receptor [sIL-2r] and angiotensin converting enzyme [ACE] were not suggestive for sarcoidosis.

The follow-up lumbar puncture [see Table 1] noted pleocytosis with now predominantly lymphocytes. Total CSF-protein levels decreased from 2470 mg/l to 1510 mg/l. The total CSF cell count showed an increase to 360 cells – Fluorescence Activated Cell Sorting [FACS] analysis was used to ruled out clonal expansion of hematological cells in the CSF and cytologic analysis did not detect any malignant cell formations.

**Figure 1:**
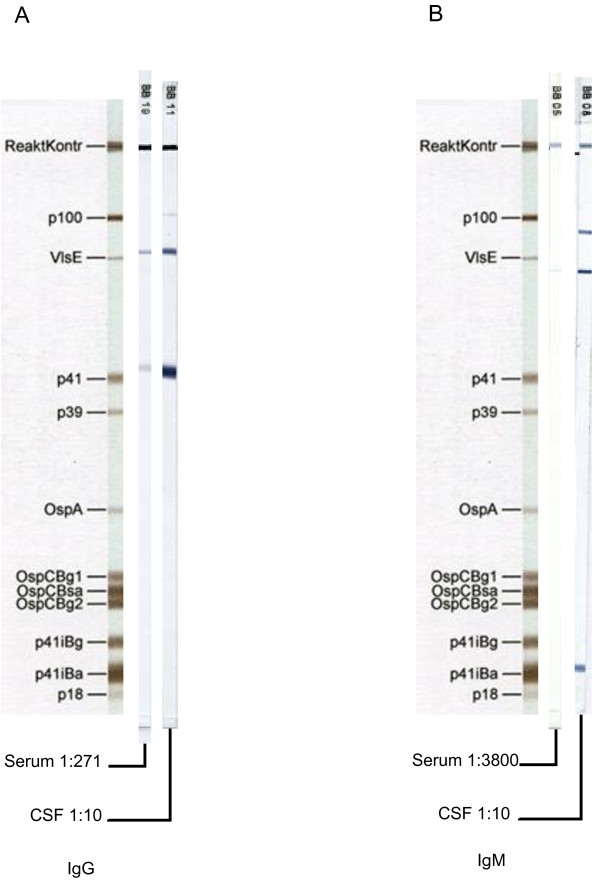
**IgG lineblot [recomBlot, Mikrogen]; Serum 1:271 dilution; CSF sample, 1:10 dilution.** Both samples with equal total IgG-concentrations (27 mg/l). Figure 1B IgM lineblot [recomBlot, Mikrogen]; Serum sample diluted 1:3800; CSF sample diluted 1:10. Both samples with equal total IgM-concentrations (0.392 mg/l). Results from CSF analysis were positive for borrelia specific intrathecal IgG antibody production. The intensity of the reactive IgG and IgM bands [Optical Density, OD] was significantly stronger in the CSF sample than in the serum and the specific antibody index [AI] was strongly positive with 20.6.

In view of lymphocytic CSF pleocytosis and facial palsy, Borrelia serology was repeated. Now the Borrelia specific CSF/serum IgG-antibody index (AI) was clearly positive, valued 20.6. The intrathecal Borrelia specific antibody production was further confirmed by IgG- and IgM-adjusted Immunoblots based on recombinant antigens (recomBlot, Mikrogen, Martinsried, Germany). Here, for both, IgG and IgM, clearly stronger reactive bands were visible in the CSF compared to serum (Figure 1). Routine PCR from CSF targeting *ospA* of *B. burgdorferi* was negative. All three criteria required in current guidelines for the definite diagnosis of LNB were met [see Table 2.]: I. neurological symptoms suggestive for LNB, II. CSF pleocytosis and III. *B. burgdorferi* specific intrathecal antibody production [1]. At this point antiinfective treatment was adjustet to i.v. monotherapy with ceftriaxone 2 g q24 for 21 days.

**Table 2:** **Criteria required for the definite diagnosis of LNB [all three criteria fulfilled], possible diagnosis of LNB [two of three criteria fulfilled]**[1]

**Criteria**	**Patient symptoms in case report**
neurological symptoms suggestive for LNB	Peripheral paresis of the VII. cranial nerve
CSF pleocytosis	Pleocytosis in all three CSF samples, initially predominatly granulocytes, then lymphocytic pleocytosis
Specific intrathecal antibody production, positive AI	AI highly positive at 20.6

For an additional diagnostic workup, specimens of the first two lumbar punctures and a third follow-up CSF sample were further investigated at the German National Reference Centre for Borrelia. Levels for CXCL13 were markedly elevated in the first two CSF samples, i.e. 5000 pg/ml and 2000 pg/ml. The cut-off range of the assay used is set at 100 - 250 pg/ml according to recent studies that reported sensitivities between 88% and 98% and specificities between 89% and 98% [4,5]. In the third liquor sample, after the patient received appropriate antbiotic therapy, CXCL13 levels have already been decreased to 450 pg/ml [see Table 1 and CSF cell count dropped to 190 cells. However, cranial nerve palsy was persisting, without signs of clinical improvement. Advanced in-house OspA and p41 PCR diagnostics with several highly specific primers were positive in the first CSF specimen. Amplification and sequencing of the complete OspA gene identified *B. garinii* OspA-type 7, an overall rarely detected subtype, as the causative agent.

Immediately after the completion of antibiotic treatment the patient felt good without meningeal signs and the peripheral facial palsy was slowly improving. Two month after being discharged, a follow up lumbar puncture [see Table 1.], revealed slowly decreasing levels of intrathecal Borrelia-specific antibody production and a CXCL13 at 52.8 pg/ml, now clearly below the threshold for LNB. There were no clinical signs of cranial nerve palsy left.

## Discussion and literature review

The chemokine CXCL13 is a promising biomarker for early untreated LNB [3,6,7]. Here we report on one of the first prospectively monitored cases with confirmed LNB. There are several retrospective studies showing elevated levels of CXCL13 in patients with untreated early LNB even before Borrelia-specific antibodies could be detected intrathecally. To prove intrathecal production of Borrelia-specific antibodies, calculations that consider blood/CSF-barrier dysfunctions [AI] are used. The first prospective study on the role of CXCL13 was published recently, indicating excellent sensitivity [94.1%] and specificity [96.1%] in the diagnosis of LNB using a different CXCL13 assay at a threshold value of 1.229 pg/ml for CXCL13 in the CSF. In comparison AI was less sensitive than CXCL13 [94.1% vs. 85.7%], whereas specificity of both markers was almost equal [3]. However, only 4 patients with untreated LNB had prospectively been enrolled, 10 patients pretreated and additional 13 patients had only been retrospectively analyzed [3].

CXCL13 is measurable in early untreated LNB even before borrelia-specific intrathecal antibody production has started. The hypothesis for this finding is a sequential chain of events that is assumed to happen before intrathecal antibody production starts: After invading the CNS, spirochetes interact with human monocytes, macrophages and dendritic cells, where CXCL13 production is induced via toll-like receptor 2 [TLR-2] activation [8]. CXCL13 then attracts and activates B-cells, which migrate into the CSF and only subsequently start producing (specific) intrathecal antibodies. In pretreated patients, by contrast no or only minimal elevations of CXCL13 levels were observed, pointing to a rapid downregulation of CXCL13 production after specific antibiotic therapy is initiated [9]. Our results match this hypothesis perfectly. In the first CSF a granulocytic pleocytosis was detected, indicating a very early phase of neuroborreliosis as aseptic meningitis [10], that already presented with very high levels of CXCL13, which dropped to only 40% of the initial value within three days. Therefore our results are consistent with earlier reports showing CXCL13 decreases under appropriate anti-infective treatment, suggesting the elimination of the causative bacterial agent. The fact that CXCL13 has dropped during the first days of appropriate treatment, while the CSF cell-count further increased, leads us to the presumption that CXCL13 measurements might be superior to follow-up cell-count examinations in this respect. However, in current guidelines [1] for the treatment of LNB no recommendation for routine use of CXCL13 is noted. CXCL13 is a promising new biomarker, but further research is needed to determine whether different species of Borrelia affect the CXCL13 response equally [6]. It has been shown, that *B.afzelii* induces CXCL13 production in a similar manner [11]. There are only limited data yet available regarding this aspect. In Europe LNB is predominantly evoked by *B. garinii*[3,12], whereas in North America *B. burgdorferi* sensu stricto is the only human pathogenic species, which has different clinical and neuroinflammatoric features from *B. garinii*. Here we demonstrate that, regarding *B. garinii* - at least CNS-infection with OspA type7 - leads to a strong upregulation of CXCL13. Other CNS prone spirochetes like *Treponema pallidum* induce similar CXCL13 levels in acute untreated CNS infections. Previous studies also showed, that CXCL13 might be an useful marker for neurosyphilis, particularly in HIV positive patients with a negative VDRL test [5,13]. However, CXCL13 levels in bacterial or viral meningitis mostly remain under the cutoff [3,13]. Primary and secondary CNS malignancies, like meningeal carcinomatosis, multiple sclerosis, as well as *Cryptococcus neoformans* CNS infections and congenital toxoplasmosis may lead to CXCL13 levels as noticed in untreated LNB [5].

A second interesting aspect of the current case is anti-OspC antibody negativity. OspC is an essential outer surface protein [Osp] for the establishment of the infection and dissemination of the pathogen in humans [14]. The commercial Borrelia immunoblot used in our routine diagnostics [recomBlot, Mikrogen] showed no OspC-specific bands using recombinant OspC Bg1, Bg2 or Bsa antigens. The additional IgG- and IgM-immunoblots that have been performed at the National Reference Centre for Borrelia, using further OspC variants from different strains *B. burgdorferi* sensu stricto strain B31, *B. afzelii* strain PKo, *B. bavariensis* strain PBi, *B. garinii* type strain 20047] did not show positive results, too [15]. In early disseminated Lyme Borreliosis OspC negativity is an uncommon phenomenon and *B. garinii* OspA serotype 7 is rarely isolated in LNB. In our opinion the most probable explanation for the present case is insufficient sensitivity of our tests because the respective antigen or OspC-epitope is not present in the recombinant proteins used. *B. garinii* OspA type 7 is all in all only rarely present [16]. Less than 5% of the infected ticks and *B. burgdorferi* CSF isolates from Germany are positive for OspA serotype 7 [12]. Therefore it is not surprising, that the genetically highly variable protein could not be reliably detected with our immunoblots we applied, which do not contain this specific OspC. In contrast OspA is highly conserved and is used for advanced species subtyping [17,18]. Whether recombinant antigens of rare strains should be added to conventional immunoblots for optimal diagnosis of LNB remains an open question. From an epidemiological point of view, *B. garinii* is the most common agent in LNB. There are different possibilities to explain the unusual findings in our patient, i.e. granulocytic pleocytosis, positive IgG ELISA, negative IgM ELISA test and only slightly positive immunoblot for IgM. Steroid pretreatment has been associated with a suppression of sufficient IgG production in animal models of LNB, whereas IgM production has been less affected [19]. It remains unclear, whether the aforementioned eight day prednisone pretreatment has interacted with the specific immune response in this particular case. Early CNS dissemination and evasion of the peripheral immune response is an additional hypothesis for the weak peripheral immune response compared to the strong intrathecal antibody production. Pleocytosis with predominantly granulocytes is a rare and deceptive observation. Especially in very early stages of LNB or in immunosuppressed hosts there are cases of inconsistent CSF cell count findings. *B. afzelii* might even cause LNB with normal CSF cell counts [20]. The cause of granulocytic pleocytosis and the quick shift to predominantly lymphocytes in the CSF samples of the above mentioned patient remain unclear.

## Conclusion

In conclusion, the presented case demonstrates the efficient use of CXCL13 in diagnosis and follow-up of LNB, even with initial misleading findings. Possible pitfalls in problematic cases, due to uncommon serological findings or lack of species differentiation, underscore the need for an interdisciplinary collaboration between neurologists, infectious disease consultants and microbiologists.

## Consent

Written informed consent was obtained from the patient for publication of this Case report and any accompanying figures, tables and images. A copy of the written consent is available for review by the Series Editor of this journal.

## Abbreviations

CXCL13: Chemokine C-X-C motif ligand 13; TLR-2: Toll-like receptor 2; LNB: Lyme neuroborreliosis; AI: Antibody index; ELISA: Enzyme linked immuno sorbent assay; Osp: Outer surface protein; VlsE: Variable major protein-like sequence expressed; VDRL Test: Veneral disease research laboratory test; CNS: Central nervous system; CSF: Cerebrospinal fluid; PCR: Polymerase chain reaction; IL-2: Interleukin 2.

## Competing interests

The authors declare that they have no competing interests.

## Authors’ contributions

JPB and JH have made substantial contributions to the conception and drafting of the case report. VF and CK carried out CXCL13 and all molecular and genetic studies. SM and JPB were substantially involved in the clinical decision making and interpretation of laboratory data. WVK revised critically the manuscript. All authors read and approved the final manuscript.

## Pre-publication history

The pre-publication history for this paper can be accessed here:

http://www.biomedcentral.com/1471-2334/12/344/prepub
